# Probiotics and Prebiotics as a Therapeutic Strategy to Improve Memory in a Model of Middle-Aged Rats

**DOI:** 10.3389/fnagi.2018.00416

**Published:** 2018-12-18

**Authors:** Alejandra Romo-Araiza, Gabriela Gutiérrez-Salmeán, Emilio J. Galván, Melissa Hernández-Frausto, Gabriel Herrera-López, Hector Romo-Parra, Valentina García-Contreras, Ana María Fernández-Presas, Ricardo Jasso-Chávez, Cesar V. Borlongan, Antonio Ibarra

**Affiliations:** ^1^Centro de Investigación en Ciencias de la Salud (CICSA), Facultad de Ciencias de la Salud, Universidad Anáhuac México, Huixquilucan, Mexico; ^2^Departamento de Farmacobiología, CINVESTAV Sede Sur, Mexico City, Mexico; ^3^Departamento de Microbiología, National Autonomous University of Mexico (UNAM), Mexico City, Mexico; ^4^Departamento de Bioquímica, Instituto Nacional de Cardiología, Mexico City, Mexico; ^5^Center of Excellence for Aging and Brain Repair, Department of Neurosurgery and Brain Repair, University of South Florida Morsani College of Medicine, Tampa, FL, United States

**Keywords:** associative memory, BDNF, butyrate, spatial memory, symbiotic

## Abstract

Aging is associated with morphological, physiological and metabolic changes, leading to multiorgan degenerative pathologies, such as cognitive function decline. It has been suggested that memory loss also involves a decrease in neurotrophic factors, including brain-derived neurotrophic factor (BDNF). In recent years, microbiota has been proposed as an essential player in brain development, as it is believed to activate BDNF secretion through butyrate production. Thus, microbiota modulation by supplementation with probiotics and prebiotics may impact cognitive decline. This study aimed to evaluate the effects of probiotics and prebiotics supplementation on the memory of middle-aged rats. Sprague-Dawley male rats were randomized in four groups (*n* = 13 per group): control (water), probiotic (*E. faecium*), prebiotic (agave inulin), symbiotic (*E. faecium* + inulin), which were administered for 5 weeks by oral gavage. Spatial and associative memory was analyzed using the Morris Water Maze (MWM) and Pavlovian autoshaping tests, respectively. Hippocampus was obtained to analyze cytokines [interleukin (IL-1β) and tumor necrosis factor (TNF-α)], BDNF and γ-aminobutyric acid (GABA) by enzyme-linked immunosorbent assay (ELISA). Butyrate concentrations were also evaluated in feces. The symbiotic group showed a significantly better performance in MWM (*p* < 0.01), but not in Pavlovian autoshaping test. It also showed significantly lower concentrations of pro-inflammatory cytokines (*p* < 0.01) and the reduction in IL-1β correlated with a better performance of the symbiotic group in MWM (*p* < 0.05). Symbiotic group also showed the highest BDNF and butyrate levels (*p* < 0.0001). Finally, we compared the electrophysiological responses of control (*n* = 8) and symbiotic (*n* = 8) groups. Passive properties of CA1 pyramidal cells (PCs) exhibited changes in response to the symbiotic treatment. Likewise, this group showed an increase in the *N*-methyl-D-aspartate receptor (NMDA)/AMPA ratio and exhibited robust long-term potentiation (LTP; *p* < 0.01). Integrated results suggest that symbiotics could improve age-related impaired memory.

## Introduction

Aging is associated with multiorgan degenerative changes, including those of the central nervous system; cerebral aging leads to cognitive decline that affects explicit memory and learning, which are dependent of the hippocampus (Moreno Fernández et al., 2013).

Mild cognitive impairment (MCI) is often referred as an intermediate stage between healthy aging and dementia, and it is estimated that 30%–100% of patients with MCI will evolve to the latter (Alberca Serrano and López Pousam, 2011). In fact, it has been reported that the rate of annual evolution from MCI to Alzheimer’s is 15% (Barahona et al., 2014).

MCI is associated with neuronal loss among the hippocampus, which, in turn, might be related with mitochondrial alterations and oxidative stress that leads to increased concentrations of pro-inflammatory cytokines such as interleukin (IL)-1β, IL-6 and tumor necrosis factor (TNF)-α (Mufson et al., 2012; Butterfield, 2014; Baierle et al., 2015). These changes also lead to neuroinflammation with an increased activity of microglia (Cevenini et al., 2013).

Neuroinflammation is associated with cognitive impairment and memory decline, as the hippocampus is prone to develop alterations in synaptic transmission and plasticity during inflammation (Di Filippo et al., 2013). The latter leads to poor assembly of glutamate with *N*-methyl-D-aspartate receptor (NMDAR), reducing the number of such receptors within the dentate gyrus and CA1 and CA3 regions of the hippocampus (Kumar, 2011). This disruption is, in turn, associated with alterations in long-term potentiation (LTP) generation, which represents an experimental evidence of the synaptic plasticity and memory consolidation (Rosi et al., 2005; Di Filippo et al., 2013; Baierle et al., 2015). Neuroinflammation also negatively affects hippocampal gene expression of the brain-derived neurotrophic factor (BDNF), a molecule that is closely related to synaptic plasticity and, consequently, to memory consolidation (Ryan and Nolan, 2016; Mora, 2013; Moreno Fernández et al., 2013).

BDNF enhances and maintains LTP induction, therefore it plays an important role in cognitive function (Pang and Lu, 2004). Aging is characterized by a decrease in BDNF concentrations, which suggests that the retrieval of BDNF levels could have a significant impact on the salvage of cognitive impairment (Pineda-Rodriguez et al., 2017).

A rather simple way to increase BDNF levels is through butyrate, a short chain fatty acid (SCFA) that acts as a histone deacetylase inhibitor (HDACi), therefore relaxes chromatin and enhances BDNF expression in the hippocampus (Kim et al., 2009; Berni Canani et al., 2012). Butyrate may also suppress pro-inflammatory cytokines production by inhibiting nuclear factor-kappa beta (NF-κβ) activation (Franco-Robles and López, 2015). Also, butyrate can increase the expression of enzymes involved in the synthesis of glutathione (GSH), which is an antioxidant enzyme responsible for reducing hydrogen peroxide and lipid hydroperoxide, thereby decreasing oxidative stress, another neurodegenerative factor (Hamer et al., 2009; Aguilar et al., 2016; Bourassa et al., 2016).

Intestinal microbiota produces a substantial proportion of the SCFA. During aging, occurs an alteration in microbiota (dysbiosis) with a significant increase in pathological bacteria (*Proteobacterium*) at the expense of beneficial ones (*Bifidobacterium*); this leads to a decreased production of SCFA. These alterations have been related to chronic systemic inflammation and therefore neuroinflammation (Caracciolo et al., 2014).

In the last years, evidence in regards of the relationship between microbiota and the brain has suggested that the prior may influence different MCI pathophysiological pathways (Brüssow, 2013). Therefore, supplementation with prebiotics and probiotics may restore deleterious effects observed on the brain due to aging by decreasing inflammation and oxidative stress, while increasing neurotrophic factors and neuronal plasticity (Brüssow, 2013).

Probiotics are live microorganisms that at an adequate dose, exert beneficial effects on the host (FAO/WHO, 2001). Prebiotics, for their side, are non-digestible ingredients, which are fermented by the gut microbiota and selectively stimulate the growth and activity of these microorganisms, and lead to an increase in the production of SCFA (Franco-Robles and López, 2015).

Among prebiotics, fructo-oligosaccharides (FOS) are the most known and used, such as agave inulin (Franco-Robles and López, 2016). This prebiotic selectively stimulates the growth and activity of *E. faecium*, a probiotic that has shown to decrease gut pro-inflammatory cytokine concentrations, and indirectly increases butyrate concentration by cross-feeding butyrate-producing bacteria (Huang et al., 2012).

According to the above, the aim of this study was to evaluate the effects of the administration of a probiotic (*E. faecium*) and a prebiotic (agave inulin) on the memory of middle-aged rats.

## Materials and Methods

### Animals

Adult Sprague Dawley (15 months old, 450–500 g) male rats (*n* = 68) were used. Animals were supplied by Proyecto Camina A.C. and were handled according to the NIH guidelines for management of laboratory animals. All the procedures were carried out in accordance with the National Institutes of Health *Guide for the care and use of laboratory animals*, and the Mexican Official Norm on Principles of Laboratory Animal Care (NOM 062-ZOO-1999). In addition, the Animal Bioethics and Welfare Committee approved all animal procedures (ID: 201624). All experiments were designed and reported according to the ARRIVE guidelines. In order to perform euthanasia, animals were previously anesthetized by intramuscular injection with pentobarbital.

### Study Design

To evaluate the effect of supplementing with probiotics (*E. faecium*) and prebiotics (agave inulin) on middle-aged rats, we performed three experiments. The first two studied the effect of the supplementation in memory and learning, as well as on pro-inflammatory cytokines and BDNF in the brain, whereas the third experiment studied the effect of symbiotics in brain electrophysiology.

In the first experiment, we explored the effect of pro- and prebiotics on spatial memory using the Morris Water Maze (MWM) behavioral test. In the second experiment, we explored the effect of pro- and prebiotics on associative memory using the Pavlovian autoshaping test.

In both experiments determinations of BDNF, IL-1β, TNF-α, and γ-aminobutyric acid (GABA) concentration were performed in the hippocampus area using the enzyme-linked immunosorbent assay (ELISA) technique.

Based on the results of these studies, we carried out a third experiment, consisting of only two groups (*n* = 5 per group): water (control) and *E. faecium* + agave inulin (symbiotic). Herein, we evaluated hippocampal neurons’ electrophysiology responses, which include evaluation of LTP and the assessment of pyramidal cells’ (PCs) membrane properties.

### Probiotics and Prebiotics Administration

Animals were supplemented through oral gavage with: control group received water (vehicle), and the other groups received *E. faecium* (4 × 10^8^ CFU; Franz et al., 2011), agave inulin (860 mg/kg; Milla et al., 2017) or a combination of both, contained in 1 ml of water. The treatment was administered daily for 5 weeks; on the 5th week, as we continued to supplement the rats, the spatial and associative memory tests were carried out. For the electrophysiology group of rats, we supplemented them for 5 weeks before the euthanasia was performed.

### Spatial Memory

The MWM was used to assess spatial learning and memory. It consists of a circular pool with a hidden platform submerged below the water surface, which has to be found by the animal. The location of the platform can only be encoded relative to three visual cues that were positioned equidistant above the water level; thereby using spatial memory. For this study, rats were placed at different starting positions in a circular pool (diameter 120 cm) filled with water (21–22°C). Rats were trained to find a platform (diameter 10 cm), which was submerged 2 cm below the water surface and located in the south-west (SW) quadrant of the pool. Animals that were unable to perform the test were eliminated. We used a reference memory protocol with 5-day training, in which each rat performed four acquisition trials (maximal swimming time 60 s; 20 s on the platform; inter-trial interval 20 s) per day during five consecutive days. Starting positions varied in each day. All trials were recorded, and latency time, defined as the delay in finding the platform, was used as a measure for spatial learning. During the trial phase, the platform was removed and the rats were allowed to swim freely for 60 s to analyse the percentage of time spent on the target quadrant (were the platform stood) against the percentage of time spent on the non-target quadrants. Trials were recorded and analyzed using a computerized system (Smart v3.0.02 Panlab Harvard Apparatus^®^) which calculates the latency time to reach the hidden platform based on the time-tagged XY-coordinates of the rat.

### Associative Memory

Associative memory was assessed through Pavlovian autoshaping test. For this test, four experimental chambers were used (Coulbourn Instruments, Lehigh Valley, PA, USA). Each apparatus included a standard attenuation system, and one chamber had the following inner dimensions: 25 cm width, 29 cm in length, and 25 cm in height. A nose poke sensor was mounted 4 cm above the floor and 10 cm from the right and left walls. A food magazine for rat pellets (see below) was located 5 cm to the right of the lever and 3 cm above the floor. A house light was located in the right top corner, which remained turned on for the duration of the session. Computer software was used for control and recording.

#### Food-Magazine Training

Each rat was individually placed in an experimental chamber for a habituation period (≈15 min), with access to 50 food pellets (45 mg each, dustless precision pellets, BioServ, Flemington, NJ, USA) previously placed inside the food-magazine. Once the animal ate all food-pellets and presented 150 nose-pokes (as measured by a photocell) into the food-magazine, the autoshaping program was initiated. Memory was evaluated at 24 h after the acquisition phase. Conditioned responses (CRs) were registered.

#### Autoshaping Training

The autoshaping program consisted of discrete trials, each composed of the presentation of the light stimulus for 8 s (conditioned stimulus, CS) followed by delivery of a 45 mg food pellet (unconditioned stimulus, US) with an inter-trial time (ITT) of 60 s. Once the animal placed its nose inside the nose-poke in response to the CS, the trial was shortened, and the light was turned off, and a food pellet (US) was immediately delivered, followed by the ITT. The response during CS was regarded as a CR, and its increase or decrease was considered as an enhancement or impairment measure of memory, respectively (Nieto-Vera et al., 2018). The autoshaping results were reported as the percentage obtained by the ratio: number of times the animal responded to the stimulus (by touching the nose poke and getting the pellets)/total number of CS (light).

### Enzyme-Linked Immunosorbent Assay (ELISA)

Rats were sacrificed by a lethal pentobarbital injection and decapitation. Brain samples were rapidly excised and the hippocampus was dissected and weighed. The tissue was frozen at −80°C until the measurement. Afterward, tissue samples were homogenized in a buffer in a ratio of approximately 10:1 buffer-tissue weight; the homogenate was centrifuged at 14,000 *g* for 30 min and the supernatants were used for ELISA assay. Samples were run by duplicates for each ELISA according to the instructions provided by the manufacturer: BDNF (ChemiKine™ from Merck, Germany), IL-1β (Thermo Fisher Scientific Inc., Waltham, MA USA), TNF-α (BioLegend, San Diego, CA, USA) and GABA (Aviva Systems Biology, San Diego, CA, USA). Cytokines, BDNF and GABA levels in the hippocampus were normalized to the amount of total protein according to Bradford’s method, using bovine serum albumin (BSA) and protease inhibitors (Bradford, 1976).

### Fecal Butyrate Concentrations

For butyric acid concentrations, feces were collected and frozen at −80°C until measurement. Afterward, fecal samples were lyophilized and rehydrated in distilled water. pH was adjusted between 2–3 with 5M HCl, 1 ml Clorophorm was added, and samples were centrifuged (25 min at 4,000 *g*). The concentration of butyric acid was determined by gas chromatography with a GC2010 apparatus (Shimadzu, Japan) by using a DB-1701 capillary column with 30 m length, 0.25 mm inner diameter and 0.25 μm film thickness (Agilent, Santa Clara, CA, USA). Conditions were: Oven: 250°C, split ratio: 20, Colum: 95°C (Isothermal), FID: 300°C, Make up gas Helium: 4 mL/min, H2 flow: 40 mL/min, Airflow: 400 mL/min, Retention time butyric acid: 3.5–3.6 min. An external standard (Supelco™ WSFA-1 Mix, Supelco Sigma-Aldrich Co., Bellefonte PA, USA) was used for quantification of SCFA. Results were reported as delta values (after supplementation − basal values).

### Electrophysiology Recordings

#### Brain Slice Preparation

Animals were anesthetized with pentobarbital (50 mg/kg body weight, intraperitoneal) and decapitated. Brain was exposed and placed in an ice-cold sucrose solution containing (in mM): 210 sucrose, 2.8 KCl, 2 MgSO_4_, 1.25 Na_2_HPO_4_, 26 NaHCO_3_, 6 MgCl_2_, 1 CaCl_2_, and 10 D-glucose with pH 7.2–7.35 and saturated with O_2_ (95%)/CO_2_ (5%) carbogen mixture. After 30–45 s, the hemispheres were separated through the midsaggital line; the resulting blocks of tissue were glued to the plate of a VT1000S vibratome (Leica, Nussloch, Germany) and transverse hippocampal slices (385 μm thick) were obtained. The slices were transferred and maintained for 30 min at 33 ± 2°C in an incubation solution composed of (in mM): 125 NaCl, 2.5 KCl, 1.2 Na_2_HPO_4_, 25 NaHCO_3_, 2 MgCl_2_, 1 CaCl_2_ and 10 D-(+)-glucose; pH 7.3 and continuously bubbled with O_2_(95%)/CO_2_ (5%). After incubation, the slices were stabilized at room temperature at least 1 h. Individual slices were transferred to submersion recording chambers (total volume 400 μl) at least for 20 min before the beginning of the experiments. The slices were maintained at a constant flow (3.5–4 ml/min) and perfused with a standard artificial cerebrospinal fluid (ASCF) solution containing (in mM): 125 NaCl, 2.5 KCl, 1.25 Na_2_HPO_4_, 25 NaHCO_3_, 2 CaCl_2_, 2 MgCl_2_, and 10 glucose; maintained at 34 ± 2°C with the help of an inline solution heater coupled to a temperature controller (TC-324C, Warner Instruments).

#### Whole Cell Recordings

CA1 PCs bodies were localized 50–100 μm from the slice surface and identified with infrared video microscopy and differential interference contrast optics (DIC) coupled to a Nikon FN1 microscope. Patch pipettes were pulled from borosilicate glass (tip resistance of 4–7 MΩ) and filled with a solution that contained (in mM): 135 K^+^-gluconate, 10 KCl, 10 HEPES, 1 MgCl_2_, 2Na^+^-ATP, 0.3Na^+^-GTP; pH = 7.2–7.26 adjusted with HCl. Current and voltage clamp recordings were obtained with an Axopatch 1D amplifier (Axon Instruments, Palo Alto, CA, USA), digitized and sampled at 10 kHz and filtered at 5 kHz (Digidata 1440A; Molecular Devices). The off-line analyses were performed with pCLAMP10 software (Molecular Devices).

#### Synaptic Currents Measurements

Spontaneous glutamatergic excitatory postsynaptic currents were acquired in voltage-clamp mode for two continuous minutes, at −65 mV in the presence of bicuculline (10 μM). Mixed glutamatergic responses evoked at Schaffer-collaterals were used to determine the relative NMDA/AMPA composition. The AMPA-mediated component was recorded at −70 mV whereas the NMDA-mediated responses were recorded at +40 mV.

#### Extracellular Recordings

In parallel with the patch clamp recordings, extracellular recordings were performed in area CA1. For this, pipettes were pulled from borosilicate glass with resistances of 1–2 MΩ when filled with a NaCl (3 M) solution. Extracellular stimulation was performed via bipolar electrodes made of nichrome wire (38 μM bare diameter) and the stimulation electrode was positioned in the stratum radiatum, on the opposite side to the recording pipette (120 ± 25 μM distance). Test stimuli (0.06 Hz) consisted of paired monopolar pulses (10 ± 5 μA intensity; 100 μs duration) that evoked 30%–50% of the maximal response. This value was previously determined with a series of Input-Output (I-O) curves. The test stimuli were delivered with a high voltage isolator unit (A365D; World Precision Instruments, Sarasota, FL, USA), controlled with a Master-8 pulse generator (AMPI, Israel). The responses were amplified with a Dagan BVC-700A amplifier (Minneapolis, MN, USA) coupled to an extracellular headstage (Dagan 8024) and high-pass filtered at 0.3 Hz. Additional electrical noise suppression was achieved with a Humbug noise eliminator (Quest Scientific Instruments, North Vancouver, BC, Canada). The evoked responses were displayed on a personal computer-based oscilloscope and digitized for storage and off-line analysis with custom-written software (Lab View system, National Instruments, Austin, TX, USA).

#### Stimulation Protocols

Induction of LTP was obtained with high-frequency stimulation (HFS) that consisted of 100 pulses delivered at 100 Hz, repeated twice with a 10 s interval.

### Statistical Methods

Statistical analysis was performed using Prism 5 software (Prism 5.01, GraphPad Software Inc., San Diego, CA, USA). Data are expressed as Mean ± standard error of the mean (SEM). Each data set was analyzed for normality using the Shapiro Wilk normality test.

To analyze the latency time through the acquisition test in the MWM, a two-way ANOVA for repeated measures was used. For the autoshaping task, cytokine, BDNF and GABA concentrations a Kruskal-Wallis followed by Mann-Whitney U test was performed. Correlation of cytokines with MWM, was analyzed using the Spearman’s correlation coefficient (for TNF-α) and Pearson’s correlation (for IL-1β). Finally, for the electrophysiology test the Mann-Whitney U test was performed. *p* < 0.05 was considered statistically significant.

## Results

### Supplementation With *E. faecium* and Agave Inulin Enhances Spatial Memory in Middle-Aged Rats

**Figure 1:**
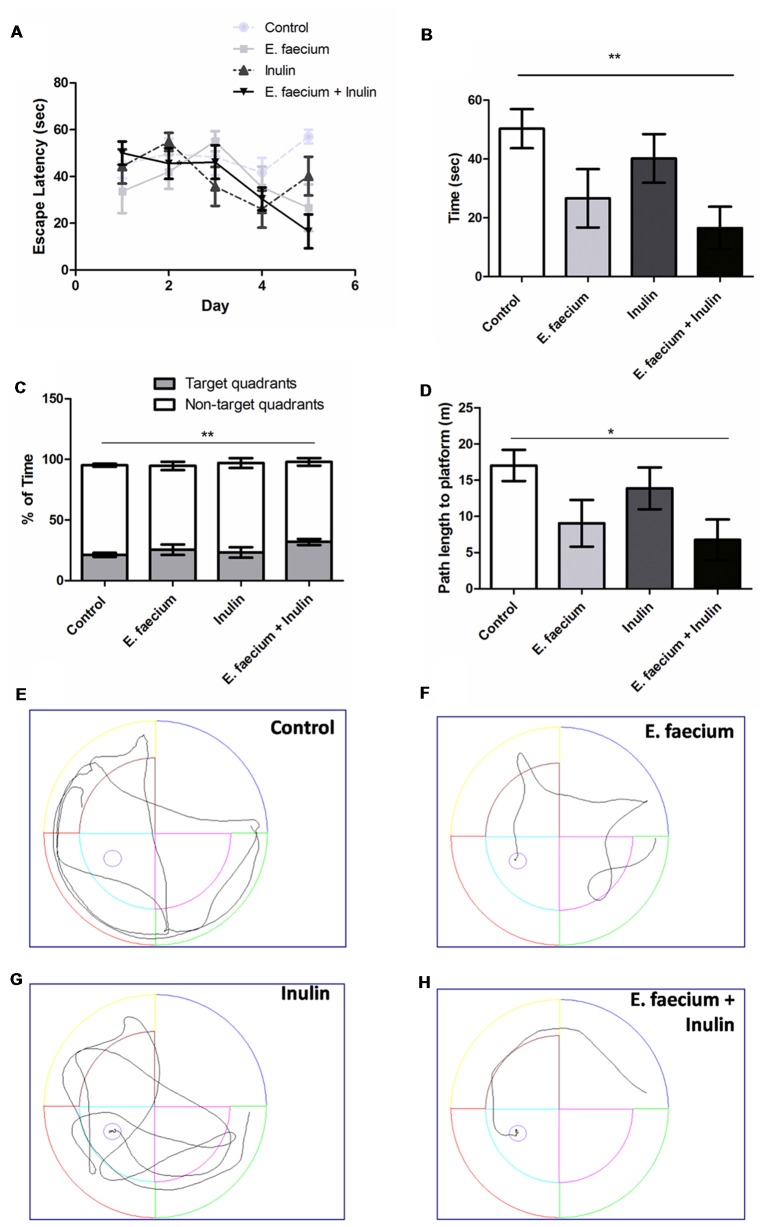
Results from the Morris Water Maze (MWM) test. **(A)** Escape latency time (seconds) from starting point in the North quadrant and platform in Southwest (SW) quadrant shows a decrease in time in all the supplemented groups. Two-way ANOVA for repeated measures followed by *post hoc* Bonferroni, *F*_(12,116)_ = 1.96; *p* = 0.03; **(B)** In day 5 of the acquisition phase, symbiotic group shows a significantly lower escape time vs. control group. Mean ± SEM, *n* = 8, Mann-Whitney U **p < 0.01; **(C)** Time spent (percentage) in the target quadrant compared to the non-target quadrants. Mean ± SEM, *n* = 8. Mann-Whitney U test; **p < 0.01; **(D)** Path length to platform (m) measured during the last day (5th) of the acquisition test. Mean ± SEM, *n* = 8, Mann-Whitney U; *p < 0.05;** (E–H)** Illustrative images of rats trajectories in MWM during last days’ trials of study groups: control **(E)**, probiotic (*E. faecium*; **F**), prebiotic (agave inulin; **G**) and symbiotic (*E. faecium* + agave inulin; **H**). This experiment is one of two in which the same effect was observed.

Spatial memory test showed a decrease in escape latency time in all the supplemented groups, the interaction between therapy and time was statistically significant (*F*_(12,116)_ = 1.96, *p* = 0.03; Two-way ANOVA for repeated measures followed by *post hoc* Bonferroni test; Figure 1A). When comparing only the last day of the acquisition phase, a significant difference was found between control and symbiotic group (50.30 ± 17.60 vs. 16.54 ± 19.09 s, respectively, *p* < 0.05; Mann-Whitney U test; Figure 1B). To strengthen these results, we calculated the Cohen’s *d*. Results show a large size effect in the spatial memory of the symbiotic group (*d* = 1.84) and in the probiotic group (*d* = 1.04); the prebiotic group showed a medium size effect (*d* = 0.49). Representative paths of the last day of acquisition phase for each group are illustrated in Figures 1E–H.

On the 6th day, memory retention was analyzed by removing the platform and comparing the time spent in the target quadrant to the average time spent in the non-target quadrants. The symbiotic group spent the longest time on the target quadrant, showing a significant difference against control and symbiotic groups (21.27 ± 1.65 and 31.86 ± 2.62% for control and symbiotic groups, respectively; *p* < 0.01; Mann-Whitney U test; Figure 1C).

We also analyzed the path length on day 5 of the acquisition test. In this case, control group traveled the most distance and symbiotic group the shortest one. The comparison among the groups showed a significant difference between control (17.04 ± 2.16 m; Mean ± SEM) and symbiotic (6.76 ± 2.82 m) groups (*p* < 0.05; Mann-Whitney U test; Figure 1D). There was no difference when comparing the control and symbiotic against inulin (13.88 ± 2.89 m; *p* > 0.05) or *E. faecium* (9.04 ± 3.23 m; *p* > 0.05). We also calculated a Cohen’s *d* and find a large effect in symbiotic and probiotic groups (*d* = 1.56 and 1.08, respectively) and a small effect in the prebiotic group (*d* = 0.45).

### Associative Memory Was Not Significantly Enhanced in Rats Supplemented With *E. faecium* and Agave Inulin

Pavlovian autoshaping test evaluates associative memory through a CS consisting of a nose poke sensor that provokes food pellets to be immediately delivered.

**Figure 2:**
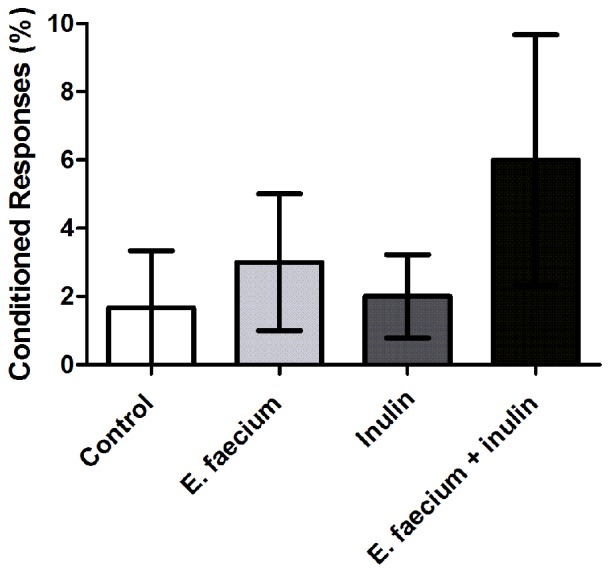
Results from Pavlovian autoshaping test. Conditioned responses (CRs) at 24 h after a training session. Results are presented as Mean ± SEM; *n* = 5; Kruskal-Wallis test (*ns*). This experiment is one of two in which the same effect was observed.

Figure 2 shows that animals supplemented with symbiotics presented a higher (although non-significant; *p* = 0.79; Kruskal-Wallis test) percentage of CRs at 24 h after the training session (Figure 2). We also calculated a Cohen’s *d* for this data set. Results show a medium size effect of symbiotics on learning-associative memory at 24 h (*d* = 0.78).

### Supplementation With *E. faecium* and Agave Inulin Decreases Pro-inflammatory Cytokines in the Hippocampus of Middle-Aged Rats

After the cognitive tests (MWM and autoshaping), we evaluated molecular aspects that would suggest possible pathways associated with the beneficial effect of the probiotics and prebiotics observed on learning and memory. Several studies have reported that neuroinflammation is a possible cause of neuronal death and consequently of cognitive decline. Microbiota has an immunomodulatory effect (through the release of SCFA) that could decrease pro-inflammatory cytokines (Caracciolo et al., 2014). Thus, we assessed the amount of IL-1β and TNF-α in the hippocampus.

Cytokine concentrations in the hippocampus were lower in the three groups that received pro-or/and prebiotics (Table 1). The symbiotic group had the lowest pro-inflammatory cytokines levels, being TNF-α the lowest one with a significant difference against control (574.3 ± 36.98 vs. 1352 ± 92.19 pg/μg of protein; *p* < 0.0001; Mann-Whitney U test). On the other hand, both symbiotic and probiotic groups had a lower IL-1β concentration which was significantly different to the one observed in the control group (356.5 ± 21.9 and 365.1 ± 25.64 for symbiotic and probiotic group, respectively, vs. 463.4 ± 20.26 pg/μg of protein for control group, *p* < 0.05 for both pairwise comparison (symbiotic vs. control or probiotic vs. control), Mann-Whitney U test).

**Table 1:** Cytokines concentrations in the hippocampus of middle-aged rats that performed Morris Water Maze (MWM; pg/μg of protein).

	Control (Water)	Probiotic *(E. faecium)*	Prebiotic (Inulin)	Symbiotic (*E. faecium* and inulin)
IL-1β	463.4 ± 20.26	621.4 ± 159.5	365.1 ± 25.64*	356.5 ± 21.9*
TNF-α	1352 ± 92.19	1384 ± 128.6	851.2 ± 122.6*	574.3 ± 36.98***

*p < 0.05; ***p < 0.0001 vs. control—Kruskal Wallis followed by Mann-Whitney U test. This experiment is one of three in which the same effect was observed.

Some studies have shown that an increased level of pro-inflammatory cytokines, especially IL-1, affects animals’ performance in memory tests such as MWM; for this reason, we performed a correlation analysis between cytokine levels and memory test outcome.

**Figure 3:**
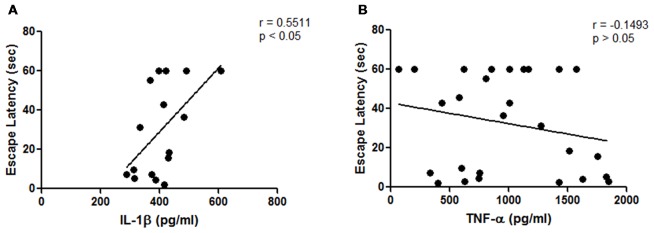
Results from hippocampal cytokines concentrations of middle-aged rats. **(A)** Correlation analysis between hippocampal interleukin-1β (IL-1β) concentrations and escape latency time on the last day of the acquisition phase (*p* < 0.05; Pearson’s correlation, *n* = 3 animals for *E. faecium* group, four animals for each control, inulin and symbiotic groups); **(B)** Correlation analysis between hippocampal tumor necrosis factor-α (TNF-α) concentrations and escape latency time on the last day of the acquisition phase (*p* = 0.2066, Spearman’s correlation, *n* = 8 animals per group).

Therefore, we analyse the correlation between cytokines and escape latency time on the last day of acquisition phase. Results showed a better performance in MWM when cytokine levels were low (Figure 3), however only IL-1β shows a statistically significant correlation (*r* = 0.5511; *p* < 0.05; Pearson’s correlation test).

To reinforce our results, the concentrations of cytokines in the hippocampus of the second experiment were also evaluated (Pavlovian autoshaping test). In this regard, the same results were observed on cytokine levels. The symbiotic group had the lowest pro-inflammatory cytokine levels, being TNF-α the lowest one with a significant difference against control group (733.6 ± 33.74 vs. 1518 ± 302.4 pg/μg for symbiotic and control groups, respectively; *p* < 0.01; Mann-Whitney U test). TNF-α was also lower in the group supplemented with agave inulin (934.7 ± 175.7 vs. 1518 ± 302.4 pg/μg for inulin and control groups, respectively; *p* < 0.05; Mann-Whitney U test).

IL-1β was significantly lower in the symbiotic group compared to the control one (193.8 ± 31.24 vs. 583 ± 90.24 pg/μg for symbiotic and control groups, respectively; *p* < 0.01; Mann-Whitney U test; Table 2).

**Table 2:** Cytokines concentration in the hippocampus of middle-aged rats who performed the Pavlovian autoshaping test (pg/μg of protein).

	Control (Water)	Probiotic *(E. faecium)*	Prebiotic (Inulin)	Symbiotic (*E. faecium* and inulin)
IL-1β	583 ± 90.24	440.6 ± 71.56	435.1 ± 89.91	193.8 ± 31.24**
TNF-α	1518 ± 302.4	1108 ± 225.8	934.7 ± 175.7*	733.6 ± 33.74**

*p < 0.05; **p < 0.01 — Kruskal Wallis followed by Mann-Whitney U test. This experiment is one of three in which the same effect was observed.

In this experiment, correlation analysis between cytokine concentrations and CRs percentage was also analyzed. There was no correlation found at 24 h after training.

### BDNF Concentrations Are Increased in the Hippocampus of Rats Supplemented With *E. faecium* and Agave Inulin

**Figure 4:**
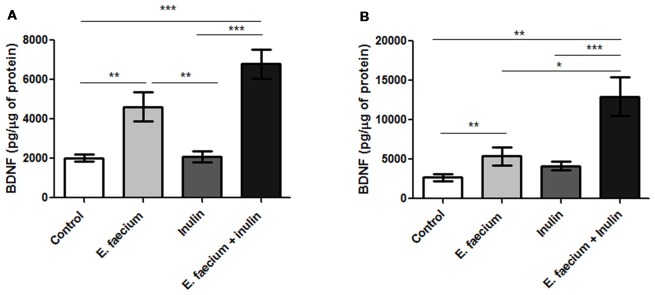
Results from hippocampal brain-derived neurotrophic factor (BDNF) concentrations of middle-aged rats. **(A)** Rats who underwent to MWM task. BDNF concentrations were significantly higher on the hippocampus of the symbiotic group as compared to the control one. Mean ± SEM, *n* = 8 for each group, Mann-Whitney U, **p < 0.01, ***p < 0.0001; **(B)** Rats who underwent the Autoshaping task. Mean ± SEM, *n* = 5 for each group, Mann-Whitney U, *p < 0.05, **p < 0.01, ***p < 0.0001. This experiment is one of three in which the same effect was observed.

To investigate more mechanisms involved in the positive effect produced by the symbiotic, we now analyzed the concentration of BDNF in the hippocampus of studied animals. In the case of rats that performed the MWM test, the symbiotic group presented the highest concentration of BDNF which was significantly different to the one observed in animals of the control group (6788 ± 725.4 vs. 2013 ± 185.3 pg/μg of protein for symbiotic and control group, respectively; <0.0001; Mann-Whitney U test; Figure 4A).

This analysis was also performed in the rats that went over the Pavlovian autoshaping test. BDNF hippocampal concentrations followed a pattern similar to that observed in the rats performing MWM. The concentration reached a significant increase in the symbiotic group (Figure 4B).

### Increased Production of Butyrate Could be the Cause of IL-1, TNFα and BDNF Changes

**Figure 5:**
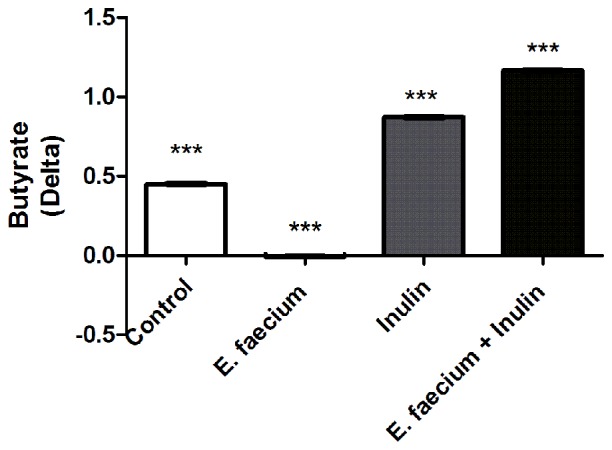
Butyrate in fecal samples of studied rats. Delta of concentration in each experimental groups. One-way ANOVA followed by Tukey’s *post hoc*, *F*_(3,12)_ = 902.6, ***p < 0.0001 between all groups. This experiment is one of two in which the same effect was observed.

Because butyrate is capable of influencing the levels of pro-inflammatory cytokines and BDNF (Sun et al., 2015), we now predicted that symbiotic-supplemented animals would present an increase in butyrate concentrations. Thus, in a subsequent step, we measured the levels of butyrate in fecal samples of the experimental animals. Our results revealed a significant increase in the absolute concentration of butyrate after inulin and symbiotic supplementation. Results are reported as delta values (after supplementation − basal value) as follows: 0.45 ± 0.008 for control, −0.008 ± 0.009 for *E. faecium*, 0.87 ± 0.008 for Inulin and 1.17 ± 0.01 for symbiotic group; One-way ANOVA followed by Tukey’s *post hoc*; *F*_(3,12)_ = 902.6, *p* < 0.0001, Inulin and symbiotic vs. control; Figure 5).

In addition to butyrate, the GABA has also be associated with an increase in BDNF (Fukuchi et al., 2014).

**Figure 6:**
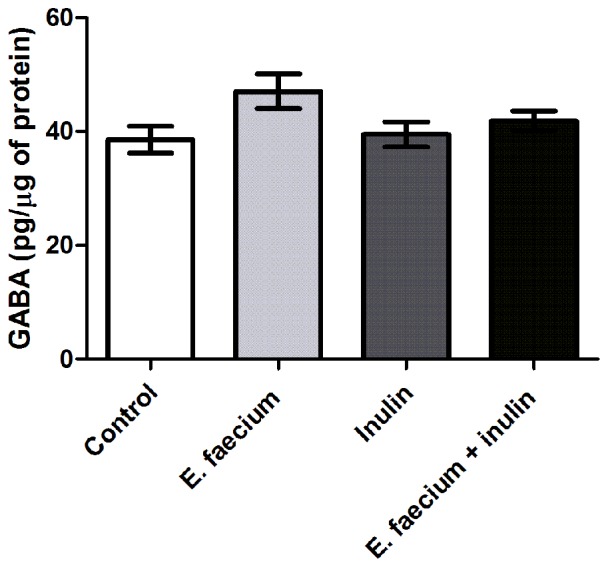
Results from hippocampal γ-aminobutyric acid (GABA) concentrations of middle-aged rats. GABA concentrations show no difference between groups. Mean ± SEM, *n* = 8 for each group, Kruskal-Wallis.

Probiotics, such as *E. faecium*, have shown to produce GABA from glutamate. Therefore, we intended to elucidate if GABA was also increased in those groups supplemented with this probiotic (those subjected to MWM), since, it could also be influencing the increment of BDNF in the hippocampus. Figure 6 shows that there was not a significant increase of this neurotransmitter in groups supplemented with *E. faecium* (*p* = 0.1407; Kruskal-Wallis test).

### Supplementation With *E. faecium* and Agave Inulin Modifies Passive Properties, the NMDA/AMPA Ratio and Facilitates Induction of LTP in Area CA1

#### Passive and Active Properties of CA1 Pyramidal Cells

**Figure 7:**
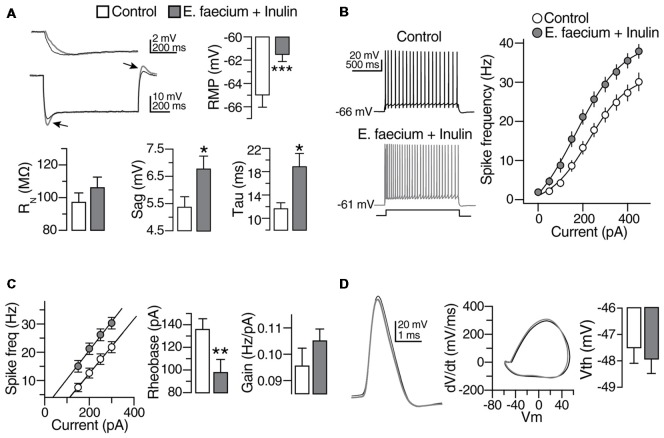
Subthreshold modulation of membrane properties of CA1 pyramidal cells (PCs) following *E. faecium* + Inulin supplementation. **(A)** Upper panels. Bar graphs depicting changes in resting membrane potential (RMP), Input resistance (R_N_) and membrane time constant (ms) in control (empty bars) and from treated animals (gray bars). Bottom panels compared to control, treated animals exhibited increased “sag” conductance (see arrowheads). Right panel, examples of changes in the membrane time constant and sag conductance in the CA1 PCs from treated animals (gray traces; measurements were performed in 43 cells/8 animals for control condition and 45 cells/8 animals for the symbiotic group). **(B)** Scatter plot and representative voltage traces summarizing the changes in the action potential frequency obtained with different current injections in control and CA1 PCs from treated animals (*n* = 21 cells/8 animals for control condition and 23 cells/8 animals for the symbiotic group). **(C)** Bar graph depicting the reduction in the rheobase current required to elicit action potentials after the symbiotic supplementation. Right panel summarizes the increase in the gain of CA1 PCs of *E. faecium* + Inulin treated animals (*n* = 21 cells/8 animals for control condition and 23 cells/8 animals for the symbiotic group). **(D)** Superimposed action potential waveforms (left panel), action potentials phase plots (middle panel) and bar graphs of the action potential threshold (right panel) showing that the supplementation with symbiotics does not alter the action potential kinetics. Gray traces and bars belongs to CA1 PCs from treated animals. *p < 0.05, **p < 0.01, ***p < 0.001 or higher statistical significance.

Based on our previous findings, in the next series of experiments we only contrasted symbiotic group vs. the control one (with no treatment); we sought to determine whether the supplementation with symbiotics modifies the passive and active properties of CA1 PCs of the hippocampus. Hippocampal slices were prepared (see “Materials and Methods” section for details) and whole cell recordings in voltage and current clamp were performed. Compared to control, CA1 PCs from animals treated with *E. faecium* + Inulin (see Figure 7A) exhibited a more depolarized resting membrane potential (RMP; control RMP −65.28 ± 0.6; treated animals = −62.46 ± 0.6 mV; *p* < 0.05; *n* = 43 cells/8 animals for control condition and 45 cells/8 animals for the symbiotic group), without significant changes in the input resistance (R_N_) and increased membrane time constant (ms). In line with these modifications, the “sag” conductance active during hyperpolarization and at the end of the current injection exhibited a marked rebound in the experimental group. Together, these results indicate that the supplementation with symbiotics alters the electrical properties of CA1 PCs.

It is expected that modifications in passive properties of excitable cells alter its firing output. Thus, we did determine whether supplementation with symbiotics alters the firing discharge of CA1 PCs. The cells were maintained at its RMP in current clamp mode (−66 ± 0.2 mV for control and −61 ± 0.15 mV for the treated group), and increasing current injections (50 pA/0.2 Hz; 1 s duration) were successively applied. In the symbiotic group, the firing discharge exhibited an increase compared to control cells (Scatter plot in Figure 7B). The enhanced discharge was consistently observed at all the current injections observed (Control firing discharge with 400 pA = 27.95 ± 2.2; treated animals = 36.96 ± 2; *p* < 0.05; *n* = 21 cells/8 animals for control condition and 23 cells/8 animals for the symbiotic group). The representative traces in response to 250 pA of injected current are shown in the right panel of Figure 7B. We also compared the firing discharge of both experimental groups in cells maintained at the same membrane potential (−65 mV). Similar to the previous results, CA1 PCs from animals supplemented with symbiotics exhibited an increase in the firing output.

We also noticed that the increase in the firing discharge was accompanied with a reduction in the rheobase current, as indicated in the left panel of Figure 7C (rheobase current in control condition = 141 ± 11.4 pA; in treated animals = 102.2 ± 12 pA; *p* = 0.005; *n* = 21 cells/8 animals for control condition and 23 cells/8 animals for the symbiotic group). By computing the linear portion of the firing output response to the injected current, we determined that the firing discharge gain of the animals supplemented with symbiotics was 11 ± 1%.

The last set of experiments were aimed to determine changes in the action potential spike of animals supplemented with symbiotics. However, no modifications were detected in the AP waveform (see phase plot in Figure 7D) or the AP threshold (Bar graphs, Figure 7D).

#### NMDA/AMPA Ratio of CA1 Pyramidal Cells

**Figure 8:**
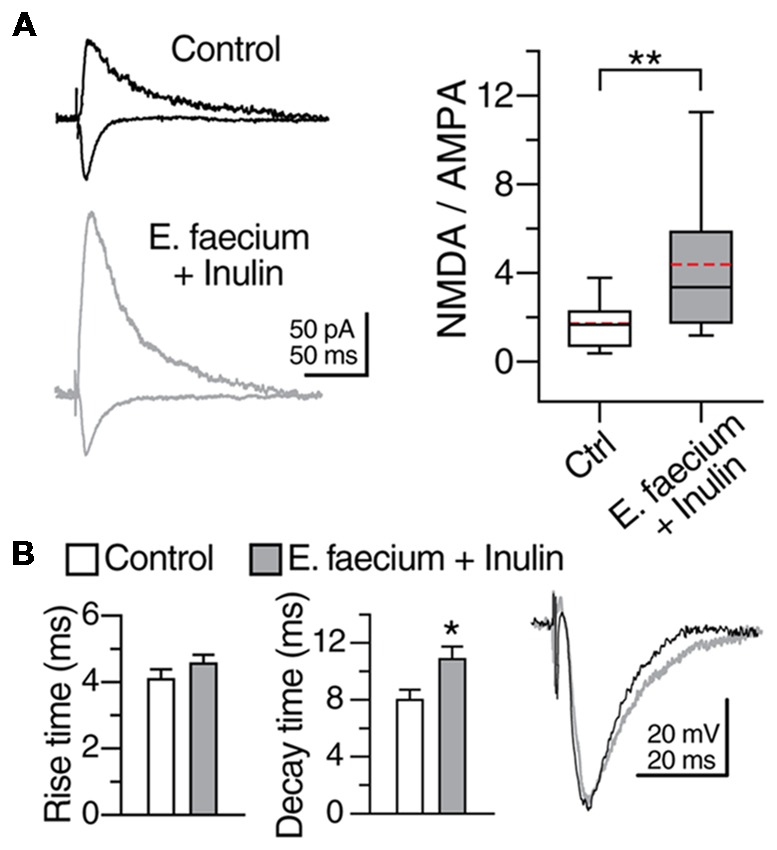
*E. faecium* + Inulin supplementation modifies the *N*-methyl-D-aspartate (NMDA)/AMPA ratio of CA1 PCs. **(A)** Representative traces (averaged from five continuous sweeps) obtained in voltage clamp mode acquired at −70 mV and +40 mV showing the typical AMPA- and NMDA-mediated response in control and in the *E. faecium* + Inulin supplemented group. The box plots in the right panel summarize the changes in the NMDA/AMPA ratio of the two experimental groups. **(B)** Bar graphs of the average Rise time and decay time of the evoked responses acquired at −70 mV in voltage clamp mode (*n* = 14 cells/3 animals for control condition and 15 cells/6 animals for the symbiotic group). The animals supplemented with *E. faecium* + Inulin exhibited an increase in the decay time of the excitatory responses. *p < 0.05, **p < 0.01.

Because the strength of the excitatory transmission at the Schaffer Collateral synapses exhibits a decline with aging, in the next series of experiments, we sought to determine whether the supplementation with *E. faecium* + Inulin (symbiotic) impacts the composition of the NMDA and AMPA glutamatergic components of the excitatory transmission (Figure 8). In this case, we only contrasted this group (supplemented) vs. the control one (with no treatment). The recordings were performed in voltage clamp mode in the presence of Bicuculline (10 μM). The AMPAR-mediated responses were recorded at −70 mV while the NMDA-mediated responses were recorded at +40 mV. In control cells, the NMDA/AMPA ratio was 1.75 ± 0.3. In contrast, CA1 PCs from supplemented animals, exhibited an increase in the NMDA/AMPA ratio of 4.13 ± 0.9 (*p* < 0.05; Mann-Whitney U test; *n* = 14 cells/3 animals for control condition and 15 cells/6 animals for the symbiotic group; Figure 8A). The increase in the NMDA/AMPA ratio observed in supplemented animals reflects an increase in the NMDA-mediated response, as also suggested by the longer decay time in the glutamatergic responses recorded at −70 mV (decay time in control 7.99 ± 0.71 ms; in supplemented animals 10.85 ± 0.9 ms; *p* < 0.05; Mann-Whitney U; *n* = 14–15 cells/3–6 animals; Figure 8B).

#### Electrically Induced Long-Term Potentiation

**Figure 9:**
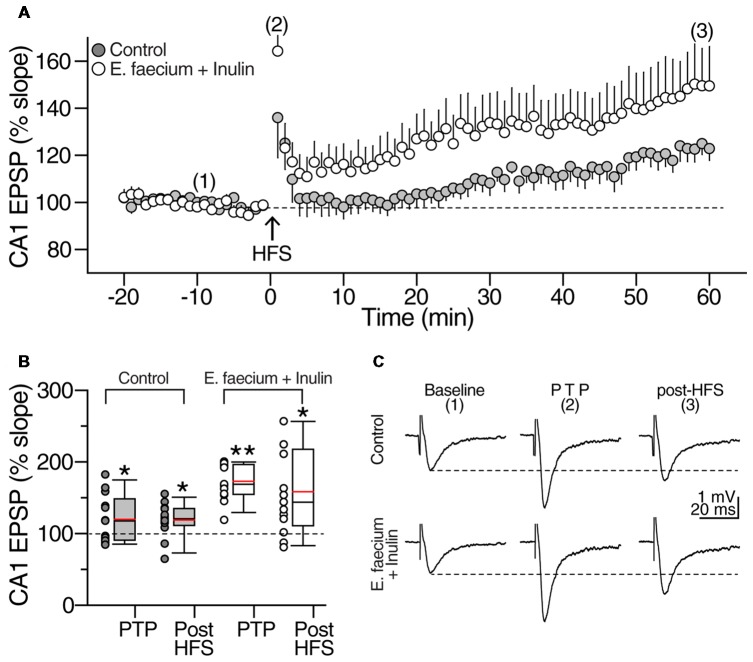
*E. faecium* + Inulin supplementation facilitates the induction of long-term potentiation (LTP) in area CA1. **(A)** Averaged time-course and magnitude of LTP of control slices (gray symbols; *n* = 13 slices/8 animals) contrasted with the responses obtained from supplemented animals (empty symbols; *n* = 13 slices/8 animals for control condition and 13 slices/8 animals for the symbiotic group). Arrowhead at time zero indicates delivery of tetanic stimulation (two 100 pulses at 100 Hz, delivered at 10 s interval). Tetanic stimulation resulted in a robust post-tetanic potentiation (PTP) of the fEPSP in both experimental groups, followed by a sustained increase in the fEPSP slope. **(B)** Box plots summarizing fEPSP changes during PTP and LTP of control (gray boxes) and *E. faecium* + Inulin supplemented group (empty boxes). The symbols at the left side of the box plots represent the average response from each experiment conducted. *p < 0.05, **p < 0.01. **(C)** Representative extracellular CA1 responses (averaged from 10 consecutive sweeps; recordings were performed in the CA1 stratum radiatum) obtained at the times indicated by the numbers in the time-course graph in upper panel **(A)**, of control (top traces) and treated animals (bottom traces).

Because the *E. faecium* + Inulin supplementation changed the AMPA/NMDA ratio at the Schaffer collateral synapses, the next series of experiments were aimed to test the synaptic capabilities of the supplemented animals. For this, we switched to extracellular recordings (see “Materials and Methods” section for details). After stabilization of the evoked response, a 20 min baseline of fEPSPs was recorded in the stratum radiatum of area CA1, and HFS (100 pulses at 100 Hz, repeated twice at 0.1 Hz) was applied at the Schaffer collaterals. In control condition, HFS caused a transient post tetanic potentiation (PTP) followed by a stable enhancement of the fEPSP slope that lasted up to 60 min (PTP in control = 119 ± 9.1%; *t*_(12)_ = 2.006, *p* = 0.68, ns; LTP in control = 121 ± 6.5 of baseline; *t*_(12)_ = 2.567, *p* < 0.02, paired *t*-test; *n* = 13 slices/8 animals for control condition and 13 slices/8 animals for the symbiotic group; Figures 9A,B, gray symbols and boxes, respectively). In contrast, slices from animals supplemented with *E. faecium* + Inulin exhibited stronger PTP and robust LTP (PTP in supplemented animals = 172.9 ± 7.1%; *t*_(12)_ = 10.303, *p* < 0.001, paired *t*-test. LTP in supplemented animals = 158.23 ± 16.8% of baseline; *t*_(12)_ = 3.407, *p* < 0.005, paired *t*-test. *n* = 13 slices/8 animals. Figures 9A,B, empty boxes). The *E. faecium* + Inulin supplementation increased the PTP response (PTP increase = 44.42%; *t*_(24)_ = 4.578, *p* < 0.0001, unpaired *t*-test) and the efficacy of LTP (efficacy increase = 33.14%; *t*_(24)_ = 2.181; *p* < 0.03, unpaired *t*-test). The representative fEPSPs obtained in control and from slices of animals supplemented with the *E. faecium* + Inulin are depicted in Figure 9C.

#### Chemically Induced Long-Term Potentiation

**Figure 10:**
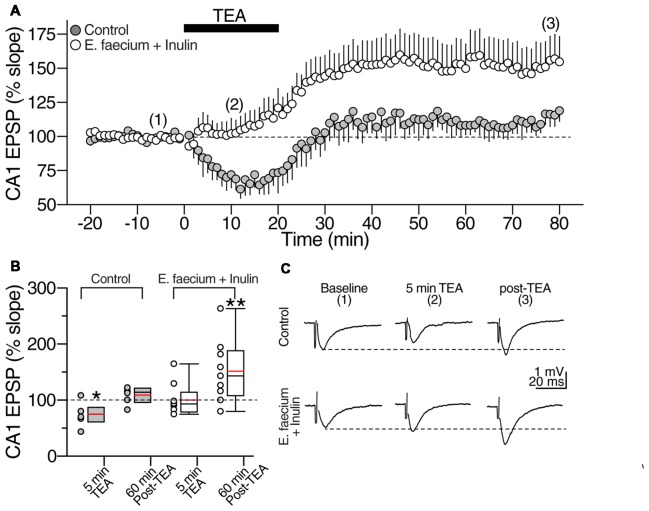
*E. faecium* + Inulin supplementation reverts the loss of synaptic potentiation induced with Tetraethylammonium (TEA). **(A)** Averaged time course graph contrasting the lack of synaptic potentiation of control slices (gray symbols; *n* = 8 cells/5 animals for control condition and 8 cells/5 animals for the symbiotic group) contrasted with the responses obtained from supplemented animals (empty symbols; *n* = 10 cells/6 animals for control condition and 10 cells/6 animals for the symbiotic group). TEA (20 mM) was bath perfused for 20 min (indicated with the filled bar) and then washed out. TEA stimulation resulted in a sustained increase in the fEPSP slope in the *E. faecium* + Inulin supplemented slices but not in control slices. **(B)** Box plots summarizing fEPSP changes at 5 min of TEA perfusion and 60 min post-TEA in control (gray boxes) and *E. faecium* + Inulin supplemented group (empty boxes). The symbols on left side of the boxes represent the average response from each experiment. *p < 0.05, **p < 0.01. **(C)** Representative extracellular CA1 responses (averaged from 10 consecutive sweeps; recorded in the CA1 stratum radiatum) obtained at the times indicated by the numbers in the time-course graph in upper panel **(A)**, of control (top traces) and supplemented animals (bottom traces).

The transient perfusion of the potassium channel blocker tetraethylammonium (TEA) induces a different form of LTP that primarily depends on the activation of voltage-dependent calcium channels rather than NMDA receptors activity (Bastos et al., 2017). Thus, in the next series of experiments, we tested whether the supplementation with *E. faecium* + Inulin influences the induction of this form of hippocampal plasticity. A stable 20 min baseline of fEPSP was acquired followed by extracellular perfusion of TEA (25 mM) for 20 min. After this, TEA-washout recording continued up to 70 min. In control condition, TEA perfusion caused transient depression of the evoked CA1 fEPSP followed by a non-significant enhancement of the evoked response (control fEPSP during TEA perfusion = 74.4 ± 8.6% of baseline; at 80 min washout = 108.7 ± 6% of baseline; *t*_(7)_ = 1.253, *p* = 0.26, paired *t*-test; *n* = 8 slices/5 animals for control condition and 8 slices/5 animals for the symbiotic group; gray symbols in the time-course graph in Figure 10A and gray box plots in Figure 10B). In contrast, the evoked responses from animals supplemented with *E. faecium* + Inulin exhibited a sustained increase of the fEPSP slope during TEA perfusion that continued throughout washout (fEPSP slope in supplemented animals during TEA perfusion = 99.9 ± 1.6% of baseline; at 80 min washout = 151.18 ± 18.6% of baseline; *t*_(9)_ = 2.776, *p* = 0.02, paired *t*-test; *n* = 10 slices/6 animals for control condition and 10 slices/6 animals for the symbiotic group; Time-course graph in Figure 10A and empty box plots in Figure 10B). Thus, the *E. faecium* + Inulin supplementation increased the efficacy of the chemically-induced LTP in 39.06%; however, the increase in the efficacy did not reach statistical significance (*t*_(16)_ = 1.796, *p* = 0.095; unpaired *t*-test). The representative traces in control condition and from animals supplemented with *E. faecium* + Inulin are depicted in Figure 10C.

## Discussion

Cognitive impairment in aging has been mainly associated with brain inflammation but also to a singular deficit of BDNF, an important neurotrophic factor participating in memory and learning. Regarding the first, it is known that aging is linked to a state of chronic systemic inflammation that can lead to neuroinflammation, which in turn has been linked to MCI. Overexpression of pro-inflammatory cytokines, particularly IL-1β, has shown to significantly affect spatial memory tasks and, is associated with both dementia and delirium (Simen et al., 2011). Likewise, high concentrations of IL-1β in the hippocampus affect late-phase LTP and, therefore, may also impair memory consolidation (Skelly et al., 2018).

Peripheral cytokine levels also appear to affect working memory. Systemic inflammation with a predominance of IL-1β has been associated with altered brain functions, since it can produce an irreversible depolarization of the membrane and lead to pro-convulsive activity and neuronal death (Skelly et al., 2018). Together, the above findings support the idea that a continuous and progressive inflammatory state in the brain impairs cognitive function. That is why, therapeutic strategies directed to prevent or diminish cognitive impairment should contemplate the reduction of inflammation. In line with this, the present study showed that symbiotic therapy was able to diminish inflammatory markers like IL-1β and TNF- α, in addition, the decrease of this cytokines significantly correlated with a better performance in memory and learning tests. These favorable effects were likely induced —at least in part— by the anti-inflammatory effect induced by the symbiotic supplementation. This therapy could be exerting anti-inflammatory actions through different pathways. As was shown in the present study, symbiotic therapy reinforced the production of butyrate a SCFA with anti-inflammatory properties that also influences the production of BDNF (Sun et al., 2015; Bourassa et al., 2016). SCFA, can bind to the G protein-coupled receptor 43 (GPR43, a receptor strongly related to inflammatory diseases) and regulate the production of inflammatory cytokines in mononuclear cells.

On the other hand, inulin by itself also plays an important role as an anti-inflammatory factor since, it could counteract the expression of GPR43. Inulin as well can act as signaling and immunomodulator, since it may mimic pathogen-associated molecular patterns (PAMP) and bind as ligand in Toll-like receptors (TLR), which are crucial proteins with immunomodulatory effects, thus increasing IL-10 (an anti-inflammatory cytokine) in mononuclear cells of human peripheral blood. Likewise, inulin could stimulate the activation of the AMP-activated kinase (AMPK), which inhibits the NF-κB, a transcription factor related to TLR4 and inflammatory processes (Peshev and Van den Ende, 2014). Therefore, the anti-inflammatory effects induced by symbiotic supplement could be part of the mechanisms improving cognitive function; however, another important player is likely BDNF. The increment observed in BDNF levels in the symbiotic–treated group could also be improving cognition.

With this respect, it has been shown that, the expression of *bdnf IV* is necessary for long-term memory formation and, BDNF is required to be present before the onset of memory consolidation and during the first 24 h. BDNF plays a critical role in promoting hippocampal-neocortical interactions that can lead to memory storage (Bambah-Mukku et al., 2014). Microbiota has been linked to changes in BDNF levels, since it has been observed that mice treated with antibiotics—which leads to dysbiosis—decreases hippocampal BDNF levels (Bercik et al., 2011). Both prebiotics and probiotics have been shown to increase BDNF levels in the hippocampus. Different probiotics such as *Bifidobacterium longum* have shown to increase the concentrations of this neurotrophic factor (Leung and Thuret, 2015). FOS like inulin, have also shown to significantly increase the mRNA of BDNF in the dentate gyrus of the hippocampus; this type of fiber also promoted an increase of NR1, a subunit of the NMDAR, in the dentate gyrus (Savignac et al., 2013).

The increase in BDNF promoted by prebiotics and probiotics as shown in the aforementioned studies, has also been mainly related to the release of SCFA, mostly butyrate. Butyric acid-producing bacteria, such as *Clostridium butyricum*, have shown to improve cognitive decline and histopathological changes in the CA1 area of the hippocampus in vascular dementia (VaD) mice, which was associated with a significant increase in BDNF levels and an increase in fecal butyrate (Liu et al., 2015). Butyrate has also been shown to display anti-inflammatory effects by inhibiting NF-κB, which in turn regulates inflammatory cytokines (IL-1β, TNF-α, IL-6, among others; Berni Canani et al., 2012). Among these, IL-1β has been shown to impair BDNF signals, so the decrease in this cytokine –as was shown in this work- may also be related to the higher BDNF concentrations in the symbiotic group (Carlos et al., 2017). Although, we only measured butyrate concentration in feces, it has been demonstrated that supplementation with butyric-acid producing bacteria increase butyrate contents also in the brain (Liu et al., 2015; Sun et al., 2015).

In general, the aforementioned mechanisms (anti-inflammation and BDNF production), independently, could be improving the neurological performance and the electrophysiological outcome observed in rats supplemented with the symbiotic. This study showed that supplemented groups (either with the prebiotic or the probiotic supplement) had a better performance especially in spatial memory; however, the group receiving the symbiotic supplementation presented the best memory outcome. The spatial memory was significantly better in this group compared to the control one. Despite this finding, we did not observe the same effect in the case of associative memory. In this test, even though symbiotic-supplemented animals presented the best performance, they did not present a statistically significant difference when compared with the other groups. With this regard, it is important to mention that associative memory is not entirely dependent on the hippocampus.

For Pavlovian conditioning to occur, it is necessary that the CS precede the US by at least 70 ms, which is related to the CR of the eyelid or “blinking.” The circuit underlying the CR is mediated and controlled by the cerebellar circuit (Poulos and Thompson, 2015). Therefore, associative memory is more related to the cerebellum. That could be the reason why, in this test, the animals supplemented with the symbiotic did not present a significant effect. Additionally, the hippocampus is the structure that is mainly affected by aging, so that spatial memory may be more affected than the associative one.

Finally, it is important to mention that, when we analyzed the clinical effect using the “Cohen’s *d*” in this experiment,—a statistical assessment that evaluates the clinical efficacy of the treatment—the analysis showed a significant clinical effect for the group of animals supplemented with the symbiotic. Although this analysis frames a possible positive effect, the result should be taken with caution since the dispersion of data in this group was quite large as a consequence of an animal that presented a very high percentage of responses. Therefore, the positive result of the Cohen’s *d* could be influenced by this reason. This is a topic that should be clarified and deeply analyzed in future investigations.

Electrophysiological outcomes were also improved in symbiotic supplemented animals. With this respect, the hippocampal LTP is essential for the coding and storage of long-term spatial memories; this depends on the activation of the NMDARs. The prevention of LTP induction in the hippocampus affects spatial learning (Bannerman et al., 2014) and during aging, it is accompanied by a deterioration in the expression of the subunits that make up the NMDA receptor as well as its function (Barnes et al., 1997). Hence, an improvement in NMDA receptor function could improve MCI.

With this in mind, it is not surprising that slices of animals supplemented with symbiotics presented a more efficient LTP than the control group, since a more significant function of the NMDA receptor is observed, which is essential in the induction of LTP at the Schaffer-CA1 synapse. On the other hand, it has been reported that activation of NMDARs promotes the synthesis of BDNF and, in turn, it acts on NMDARs and increases excitatory synaptic transmission in the cortex and hippocampus (Park and Poo, 2013; Maqsood and Stone, 2016). BDNF participates in the induction and maintenance of LTP in CA1 (Diógenes et al., 2011; Katche et al., 2013). In the present study, we found a significantly higher concentration of BDNF in the hippocampus of rats supplemented with the symbiotic; this group also performed significantly better in the spatial memory test and showed signs of memory retention by showing a preference for the objective quadrant once the platform was removed. Therefore, it is plausible to postulate BDNF as a possible inductor of the observed electrophysiological outcome.

The present work shows that there is a relationship between the microbiota and the brain, and presents for the first time that the consumption of probiotics and prebiotics improves synaptic plasticity, impacting memory and learning processes; this finding is of great importance and provides for a possible clinical application and possible treatment in the MCI area. Other investigations have reported the effect of probiotics as a mechanism to improve cognitive functions in rats. *Lactobacillus helveticus* has shown a significant reduction in escape latency time in the MWM after 4 days of training (Luo et al., 2014). *Clostridium butyricum* has also shown to improve performance time compared to control group, while increasing the time spent in the target quadrant in the memory retention phase (Liu et al., 2015). The current results clearly show that with symbiotic supplementation, it is possible to improve the memory of individuals with cognitive impairment generated by the aging process.

## Conclusion

Collectively, our results suggest that prebiotics and probiotics improve learning and memory through an increase in butyrate which in turn, provoke an increase in BDNF and a decrease in pro-inflammatory cytokine concentrations in the hippocampus. The effect of pre- and probiotics is reinforced by their combination, which suggests that there is a synergistic effect that makes symbiotics a better therapeutic strategy for MCI. This effect is evidenced by LTP and NMDAR activation. The symbiotic effect of the probiotic *E. faecium* and the prebiotic agave inulin seems to improve the cognitive function of middle-aged rats.

## Author Contributions

AR-A co-designed the study and wrote the manuscript. GG-S helped with the analysis of the study and manuscript. EG, MH-F, GH-L, and HR-P carried on the electrophysiology recordings. VG-C helped with the MWM, BDNF and GABA analysis. AF-P and RJ-C carried on the fecal butyrate concentration analysis. CB: analysis and supervision of experiments. AI provided founds, co-designed the experiments, supervised and analyzed this study and manuscript.

## Conflict of Interest Statement

The authors declare that the research was conducted in the absence of any commercial or financial relationships that could be construed as a potential conflict of interest.
